# Microbiota and Neurological Disorders: A Gut Feeling

**DOI:** 10.1089/biores.2016.0010

**Published:** 2016-05-01

**Authors:** Walter H. Moos, Douglas V. Faller, David N. Harpp, Iphigenia Kanara, Julie Pernokas, Whitney R. Powers, Kosta Steliou

**Affiliations:** ^1^ Department of Pharmaceutical Chemistry, School of Pharmacy, University of California San Francisco, San Francisco, California.; ^2^ Department of Medicine, Boston University School of Medicine, Boston, Massachusetts.; ^3^ Cancer Research Center, Boston University School of Medicine, Boston, Massachusetts.; ^4^ Department of Chemistry, McGill University, Montreal, Canada.; ^5^ Weatherhead Center for International Affairs, Harvard University, Cambridge, Massachusetts.; ^6^ Consulate General of Greece in Boston, Boston, Massachusetts.; ^7^ Advanced Dental Associates of New England, Woburn, Massachusetts.; ^8^ Department of Health Sciences, Boston University, Boston, Massachusetts.; ^9^ Department of Anatomy, Boston University School of Medicine, Boston, Massachusetts.; ^10^ PhenoMatriX, Inc., Boston, Massachusetts.

**Keywords:** α-lipoic acid, autism, dementia, dysbiosis, epigenetic, exercise, fatty acids, histone deacetylase, immune system, microbiota, mitochondria, neurodegenerative, probiotics, schizophrenia, synthetic biology

## Abstract

In the past century, noncommunicable diseases have surpassed infectious diseases as the principal cause of sickness and death, worldwide. Trillions of commensal microbes live in and on our body, and constitute the human microbiome. The vast majority of these microorganisms are maternally derived and live in the gut, where they perform functions essential to our health and survival, including: digesting food, activating certain drugs, producing short-chain fatty acids (which help to modulate gene expression by inhibiting the deacetylation of histone proteins), generating anti-inflammatory substances, and playing a fundamental role in the induction, training, and function of our immune system. Among the many roles the microbiome ultimately plays, it mitigates against untoward effects from our exposure to the environment by forming a biotic shield between us and the outside world. The importance of physical activity coupled with a balanced and healthy diet in the maintenance of our well-being has been recognized since antiquity. However, it is only recently that characterization of the host–microbiome intermetabolic and crosstalk pathways has come to the forefront in studying therapeutic design. As reviewed in this report, synthetic biology shows potential in developing microorganisms for correcting pathogenic dysbiosis (gut microbiota–host maladaptation), although this has yet to be proven. However, the development and use of small molecule drugs have a long and successful history in the clinic, with small molecule histone deacetylase inhibitors representing one relevant example already approved to treat cancer and other disorders. Moreover, preclinical research suggests that epigenetic treatment of neurological conditions holds significant promise. With the mouth being an extension of the digestive tract, it presents a readily accessible diagnostic site for the early detection of potential unhealthy pathogens resident in the gut. Taken together, the data outlined herein provide an encouraging roadmap toward important new medicines and companion diagnostic platforms in a wide range of therapeutic indications.

## Introduction

In less than 100 years, noncommunicable diseases have surpassed infectious diseases as the principal cause of sickness and death, worldwide.^1^ One hundred trillion commensal microbes (including the fungal community referred to as the mycobiome)^2^ that live in and on our body constitute the human microbiome,^3^ although a recent study^4^ estimates the overall figure to be much lower. Regardless of the absolute number, the vast majority of these microorganisms live in the gut (microbiota),^5^ where they perform functions that are essential to our health and survival. They help us digest food^6^; participate in the activation of certain drugs^7^; produce short-chain fatty acids (SCFAs) that help modulate gene expression by inhibiting deacetylation of histone proteins^8–10^; generate molecules that reduce inflammation^11^; and play a fundamental role in the induction, basic development, training, and function of our immune system.^12–14^ Thus, as a whole, the microbiome becomes an integral part of our immune makeup, and is largely inherited from the mother with significant differences consequent to cesarean versus vaginal deliveries.^15–20^ Among the many roles the microbiome may ultimately play in health and disease, it mitigates against the untoward effects from our exposure to the environment by residing as a biotic barrier between us and the world around us.^1,21–25^

Neuropsychiatric disorders^26^ are on the increase globally and, of the noncommunicable diseases, stand out as a leading cause of disability.^3,8,27,28^ Accruing evidence strongly links gut dysbiosis (gut microbiota–host maladaptation) as a risk factor in a wide range of mental illnesses that include neuropsychiatric conditions,^3,29–38^ such as autism spectrum disorder (ASD)^39–46^ and schizophrenia^39,40,46–49^ among them. There are currently no drugs approved that treat the core symptoms of ASD.^50^ The pathogenic mechanisms underlying schizophrenia, a debilitating mental disorder, are unknown^51^ and drug therapies used to treat the associated psychotic symptoms have advanced little since the introduction of clozapine in 1960.^52,53^

The reported association of mental illness with digestive disturbances dates back to Hippocrates and stands as the single consistently linked comorbidity described in the medical literature from ancient times to the present.^49^ Although the genesis of our microbiome is predominantly our mother's,^18,54^ eventually our microbiome transforms into our own unique signature.^55^ Changes in the gut microbial composition and function constantly adapt to our diet,^56,57^ and the mechanistic relationships between the gut microbiota in the development of the enteric nervous system^58^ and the preservation of our metabolic health^59,60^ are only now beginning to be elucidated.

## Microbiota-Induced Epigenetics

Advances in genetic editing technologies may help clarify whether it is our genetics that control our epigenome or epigenetics that control the genome—or, more likely, the relationship between the two is mutual.^61^ There is supporting evidence to suggest that our microbiome plays a fundamental role in this relationship.^14,62,63^ Numerous studies^64,65^ show microbe-generated metabolites are intertwined with host cell biochemistry and physiology, and SCFA-mediated cell signaling is a key pathway that gut microbes use to communicate with the host.^9,44,66–68^ Acetate, propionate (propionic acid is also commonly referred to as PPA), butyrate, and pentanoate, having respectively, 2, 3, 4, and 5 carbon atoms are SCFAs (Table 1), largely produced by microbial fermentation of complex polysaccharides (starches and fibers) in the colon (longer chain aliphatic acids with 6 to 12 carbons are considered to be medium-chain fatty acids [MCFAs]). SCFAs are absorbed into the colonic epithelium where, primarily, butyrate is consumed as a preferred fuel source by colonocytes.^69–73^ Microbiota-produced SCFAs enter the bloodstream through the portal circulation of the host and/or the distal colon and are transported to recipient tissues where they are taken up and used in a variety of cellular responses, including the regulation of gene expression.^9,47,74,75^

**Table 1.** **Chemical Structures of Fatty Acids with Two to Eight Carbon Atoms**

Many brain disorders are associated with imbalances in protein acetylation levels and transcriptional dysfunction.^76^ Histone deacetylase (HDAC) inhibitors represent a promising therapeutic option to correct these deficiencies, and numerous studies using butyrate, the most potent of the SCFA HDAC inhibitors,^9,77^ demonstrate the medicinal potential of butyrate in the intervention of neurodegenerative diseases and psychiatric disorders.^26,64,76,78–85^

**FIG. 1.**
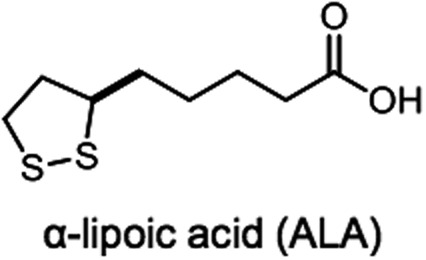
Chemical structure of *R*-(+)-lipoic acid.

α-Lipoic acid [(*R*)-5-(1,2-dithiolan-3-yl)pentanoic acid] (ALA, Fig. 1) is a naturally occurring 5-membered ring disulfide-substituted SCFA HDAC inhibitor^86,87^ with strong antioxidant activity.^88^ It is an essential cofactor in aerobic metabolism and is the central component forming the pyruvate dehydrogenase complex,^89,90^ which functionally links glycolysis in the cytoplasm to oxidative phosphorylation (OXPHOS) in mitochondria.^91^ ALA plays a role in microbial metabolism too.^92^ Although ALA is present in almost all food types that we eat,^93^ and is readily digested, absorbed, and transported to tissues, including brain,^94–96^ the amounts available from diet are low.^93^ Although the acquisition and use of ALA vary in different microbes, yeast, and animal cells,^97,98^ its functions are, nonetheless, essential to the organism, and in most prokaryotic and eukaryotic microorganisms, plant and animal mitochondria, and plant plastids, ALA is enzymatically synthesized endogenously from the MCFA, octanoate.^97–99^

In addition to its role in the metabolic pathways, ALA is reported in many research studies to be a potent activator of the nuclear factor (erythroid-derived 2)-like 2 (Nrf2) antioxidant response element signaling pathway that regulates the expression of genes whose protein products are involved in the detoxification and elimination of reactive oxygen species and electrophilic agents.^26,96,100–102^ Oxidative stress has been implicated in the pathogenesis of various neurodegenerative and neuropsychiatric disorders, including depression.^103,104^ Vasconcelos et al.^105^ showed that ALA (100 mg/kg) alone or combined with clozapine reversed schizophrenia-like alterations induced by ketamine. Ketamine is a known glutamatergic *N*-methyl-d-aspartate receptor antagonist that can induce psychotomimetic, perceptual, cognitive, and neuroendocrine responses in humans and in rodent models of schizophrenia.^106,107^ ALA also inhibits nuclear factor kappa-B (NF-κB) activation independent of its antioxidant function. NF-κB belongs to an important group of transcription factors regulated by a kinase-mediated signaling pathway that transduces signals from the cell surface to changes in gene expression.^108,109^

Fatty acids represent one of the body's long-term storage reservoirs and sources of fuel energy—the heart being a primary consumer.^110^ In the presence of respiratory oxygen, fatty acids are shuttled through the mitochondrial OXPHOS complex system, where they are degraded by two carbon units at a time to shorter-chain fatty acids (and ultimately to acetyl coenzyme A), with concomitant release of water, CO_2_, and ATP in the process. The cellular availability of SCFAs for use in epigenetic chromatin remodeling through their ability to inhibit HDAC activity, therefore, is closely tied to mitochondrial energy production and metabolism. Since both prokaryote and eukaryote cells share common pathways for energy production, for example, the citric acid cycle,^75^ it comes as no surprise that gut microbiota inexorably affect host–cell bioenergetics, which in turn fuels gene expression in the mitochondrial and nuclear genomes.^75,111^ Over a billion years of evolutionary history have allowed our mitochondrial DNA (mtDNA) and nuclear DNA to coevolve with a high degree of genetic compatibility.^112,113^ Interestingly, our microbiome and our mitogenome (mtDNA), as well as portions of our epigenome—for example, maternal silencing,^114^ are uniquely passed to each of us from our mother.

Gut microbiota have a profound influence on the host immune system.^13^ Maternal immune activation is a shared environmental risk factor for a plethora of neuropsychiatric and neurodegenerative disorders that may or may not develop into clinical symptoms in offspring.^13^ Evidence from an in-depth study^115^ of data extracted from the Danish health registry of more than 1 million children born between 1980 and 2005, focusing on cases where the mother had a viral infection with fever requiring hospitalization during the first trimester, strongly links maternal immune dysregulation with suppressed neurodevelopment and cognitive function (ASD) in their offspring.^116^ In other epidemiological studies,^117^ prenatal exposure to infection visibly stands out as a risk factor in schizophrenia and other neurodevelopmental abnormalities. The possibility that prenatal Zika virus infection from a mosquito reservoir is responsible for the current outbreak in Brazil of children born with microcephaly is a disturbing and frightening example.^118–121^

## Synthetic Biology

With a capacity to act either naturally or by manipulation, the gut microbial ecosystem is an indispensable and constituent player in the maintenance of our well-being.^7^ Thus, in treating disease, adjusting the functional composition of the gut microbiome may help facilitate and even alter the outcome of therapeutic interventions.^122,123^ Dietary sources of probiotics such as, for example, in traditional Greek yogurt, have been used since antiquity in the Mediterranean region (Mediterranean diet)^124,125^ to maintain a state of wellness. However, once pathogenic dysbiosis sets in, probiotics have not proven to be remedial^123,126,127^ and other interventional methods are being investigated. In this effort, independent work from several groups suggests that (engineered) bacteria have potential to be an effective means for delivering, enhancing, or themselves acting as therapeutic agents (“living pills”)^128^ to treat certain diseases,^22,129–132^ including psychiatric disorders,^43,48,133–136^ and significant investments are being made to adapt a variety of commensal microbial species for remodeling the gut microbiota (ecobiotics) in disease-treating indications.^133,137–139^ A similar approach aims to utilize engineered viruses to seek and selectively destroy pathogenic bacteria.^140–142^ Drawing conclusions from a study of 11 children affected by ASD that showed improvement in communication and behavioral tests after being treated with vancomycin for 8 weeks, Mangiola et al.^46^ speculate that modulation of gut microbiota through antibiotic treatment may influence the symptoms and expression of psychiatric disorders in general. Devkota^143^ takes this further by underscoring a comprehensive need for more investigations into drug–microbiome interactions and the mechanisms that are involved therein.

Interestingly, during long stays in space, the configuration of the gut microbiome of astronauts is often significantly transformed relative to the one they had on Earth.^144–146^ The ongoing NASA Twins Study, with Scott Kelly having recently returned from a historic 340-day mission aboard the International Space Station, may shine additional light on this subject.^147^ Gut microbial dysregulation can alter one's immune status and cause aberrant social and cognitive behavior.^35,148,149^ This may result in catastrophic consequences during long space flights, as for example, to Mars, if an astronaut's ability to carry out demanding tasks at a high performance and optimal level becomes severely compromised.^150^ History shows that addressing the technological challenges space exploration presents has a constructive rippling effect on the technological advances made for a wide range of applications here on Earth. Synthetic biology^151^ has potential to deliver robust and reliable organisms that can assist on long-duration astronaut missions.^152^ It is anticipated that the techniques required to be developed may also be applied to engineer phage and bacteria to explore and to therapeutically modify the gut microbiome as needed.^153^

## Brain Development and Neurological Disorders

**FIG. 2.**
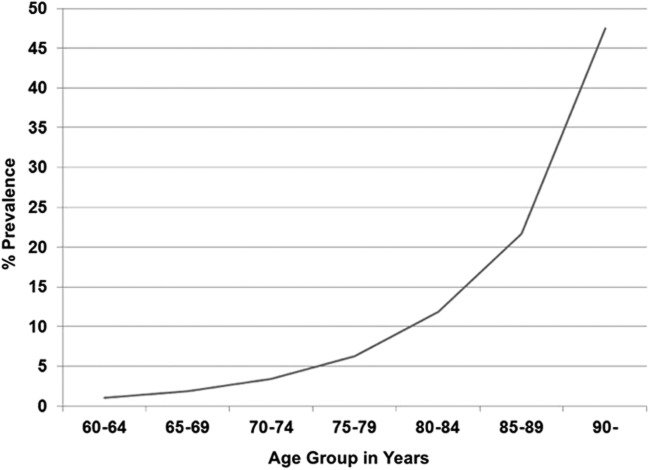
Meta analyzed estimates of dementia prevalence in the United States.

Aging is a leading risk factor (Fig. 2)^154^ in progressing to dementia.^155–159^ Although the latest studies suggest that the prevalence of dementia may be leveling off and even decreasing in some subsets of the population,^160^ for the foreseeable future, dementia will continue to be a major challenge for the healthcare establishment.^161^

The gut's evolving capacity to adapt and maintain normal microbiota, which begins at birth and continues throughout one's life, is necessary to support the metabolic activities of the brain.^59,162^ This is especially so in the early childhood years through adulthood.^59^ Studies have shown that some of the typical behavioral and physiological abnormalities associated with neurodevelopmental disorders, including autism^43–45,163–165^ and schizophrenia,^40,47–49^ can be modulated by reconfiguring the gut microbiome composition.^122,166^

Acetate, propionate, and butyrate comprise the majority of SCFAs produced in the gut by microbial fermentation.^21,69^ Propionate and butyrate can modulate brain functioning, principally appetite and energy homeostasis, through regulation of neuropeptide production.^167^ Butyrate is mostly absorbed by the colonic epithelium, whereas acetate and propionate are passed into the portal circulation.^168–170^ In gut dysbiosis, the constitutional spectrum of SCFAs varies substantially from the host's natural healthy balance,^3,32,39,60,171^ and higher than normal levels of PPA have been linked to deleterious effects on brain function^43,75,78,172–179^ in autistic children.^24,174^ Given these findings, and the fact that PPA is widely used as a food preservative,^180^ there may be cause for some concern.

Idiopathic late-onset dementia (ILOD) is characterized by a series of declining daily functional competences, most often involving memory, reasoning, and sociobehavioral abilities, in the elderly.^157,161^ Dementia encompasses a myriad of clinical symptoms typically associated with discrete neurological disorders such as Alzheimer and Parkinson diseases, hippocampal sclerosis of aging, and Lewy body and frontotemporal dementias being the more notable ones, but not collectively manifested by any one of them. Cellular processes depend on the energy supplied by their mitochondria, and dysfunctional mitochondria can lead to an unsustainable cellular bioenergetics deficit that is detrimental to the cell's function and survival.^181^ In brain cells, even a small energy deficit, which is a common occurrence during the aging process, can reduce synaptic neurotransmitter release and adversely affect synaptic function.^26,101^ Maintaining a healthy gut microbiota state is necessary to support the metabolic activities of the brain,^59,162^ and Mattson^157^ and Bourassa et al.^85^ posit that some of the common pathologies leading to ILOD and other brain disorders may be amenable to therapeutic modification by diet and lifestyle changes. For example, exercise, yoga, and meditation are lifestyle activities known to improve brain blood flow—which, presumably, can enhance perfusion of the brain with micronutrients absorbed by the gut^182^—and are increasingly being incorporated in treatments for depression and other mental disturbances.^36,127,183–185^

At the other end of the age spectrum, the correlation between impaired intellectual development and a prolonged state of malnutrition in infants and young children is inescapable.^186–189^ Recent studies have demonstrated that the normal pattern of gut microbiota assembly is disrupted in malnourished children.^162,187^ To maximize the therapeutic benefit of diet and dietary supplements, preclinical evidence suggests that a healthy microbiome in these children may need to be configured as well.^190,191^

## Concluding Remarks

The importance of physical activity coupled with a balanced and healthy diet in the maintenance of our well-being has been recognized since antiquity. However, it is only recently that characterization of the host–microbiome intermetabolic and crosstalk pathways has come to the forefront for study in therapeutic design and treatments.^68,192^ As reviewed in this report, synthetic biology has potential to develop microorganisms for correcting pathogenic dysbiosis, but this has yet to be proven. (For additional examples of the latest approaches to manipulating the microbiota, including illustrative figures, see Ash and Mueller,^193^ and articles cited therein.) In contrast, the development and use of small molecule drugs have a long and successful history in the clinical treatment of diseases. Small molecule HDAC inhibitors are already used in the clinic to treat cancer and hematological disorders,^77^ and preclinical research with SCFA HDAC inhibitors demonstrates significant potential in epigenetic treatment of neurological conditions.^26,101^ Epigenetic regulation of host–microbiota interactions by utilizing epigenomic-targeting drugs has been suggested by Alenghat and Artis.^74^

The mouth, being an extension of the digestive tract, presents a readily accessible diagnostic site for the early detection of potential unhealthy pathogens resident in the gut. Salivanomics is a rapidly emerging tool in the arsenal of salivary diagnostics.^194^ Collecting saliva or swabbing the inside cheek of the mouth is noninterventional, making it a highly attractive diagnostic procedure, particularly for infants and young children. It is not unlikely that regular visits to your dentist may soon be as important to your gut as it is to your oral hygiene, white teeth, and a nice smile.

## Abbreviations Used

ALA: α-lipoic acid
ASD: autism spectrum disorder
HDAC: histone deacetylase
ILOD: idiopathic late-onset dementia
MCFA: medium-chain fatty acid
mtDNA: mitochondrial DNA
NF-κB: nuclear factor kappa-B
Nrf2: nuclear factor (erythroid-derived 2)-like 2
OXPHOS: oxidative phosphorylation
PPA: propionic acid
SCFA: short-chain fatty acid
